# A Case of Chronic Myelomonocytic Leukemia Unmasked After Receiving J&J COVID-19 Vaccine

**DOI:** 10.7759/cureus.26070

**Published:** 2022-06-18

**Authors:** Sindhusha Veeraballi, Aditya Patel, Gowthami Are, Amr Ramahi, Sahithi Chittamuri, Hamid Shaaban

**Affiliations:** 1 Internal Medicine, Saint Michael's Medical Center, Newark, USA; 2 Hematology and Oncology, Saint Michael's Medical Center, Newark, USA

**Keywords:** covid-19 vaccine, sjogren’s syndrome, scleroderma, autoimmune disorders, chronic myelomonocytic leukemia (cmml)

## Abstract

Chronic myelomonocytic leukemia (CMML) is a clonal hematopoietic stem cell disease that comes under the overlap syndrome (myelodysplastic and myeloproliferative disorders). CMML is characterized by peripheral blood monocytosis and bone marrow dysplasia. The pathogenesis of CMML is poorly understood. Although cytogenetic and molecular abnormalities are common, they are not diagnostic. Herein, we present a rare case of CMML after receiving the J&J COVID-19 vaccine with the rare association of limited scleroderma. Based on the Surveillance, Epidemiology, and End Result (SEER) cancer statistics review 2014-2018, the five-year age-adjusted incidence rate of CMML in both sexes is 0.5/100,000, with greater incidence in males (0.7/100,000) compared to females (0.3/100,000). We emphasize the fact that, based on the previous studies reported, the association of scleroderma with CMML is very rare. Our patient had concomitant CMML and scleroderma, which were unmasked after the patient received the COVID-19 vaccine. Our case suggests the possibility of developing CMML after receiving the J&J COVID vaccine. Immunization has always been a life-saving intervention in history. As the world is foreseeing getting the COVID-19 vaccine, it is essential to report all the possible adverse events for safety monitoring. Physicians should be aware of this unusual complication of the vaccine, and more cases are needed to confirm the association between them.

## Introduction

Myelodysplastic syndrome (MDS)/myeloproliferative neoplasm (MPN) overlap syndromes are unique myeloid neoplasms with overlapping features of MDS and MPN. They consist of four adult entities: chronic myelomonocytic leukemia (CMML), MDS/MPN ring sideroblasts-thrombocytosis, BCR-ABL-1 negative atypical chronic myeloid leukemia (aCML), and MDS/MPN-unclassifiable; and one pediatric entity, being juvenile myelomonocytic leukemia [1]. CMML is characterized by peripheral blood monocytosis and bone marrow dysplasia. It occurs commonly in older adults, with a median age at diagnosis of 65 to 75 years and a male-to-female ratio of 1.5-3.1:1 [2,3]. Clinical features of CMML are non-specific; they are either related to myeloproliferative symptoms or symptoms related to cytopenias. The pathogenesis of CMML is poorly understood. Although cytogenetic and molecular abnormalities are common, they are not diagnostic [3,4]. Approximately 20% of patients have associated systemic inflammatory and autoimmune diseases (SIAD) [5-8]. Herein, we present a rare case of CMML after receiving the J&J COVID-19 vaccines, in association with limited scleroderma.

## Case presentation

A 74-year-old female with a past medical history significant for asthma, hypertension, and dyslipidemia presented to the emergency department with complaints of shortness of breath and generalized weakness for two days. The patient reports that symptoms began after receiving the first dose of the Johnson and Johnson vaccine for COVID-19. She was hemodynamically stable with a heart rate of 94, a respiratory rate of 21, and a saturation rate of 99% on room air. Physical examination was widely unremarkable except for decreased air entry with mild diffuse wheezes bilaterally on lung auscultation. No cyanosis or edema of the extremities. A complete blood count revealed hemoglobin of 9.9 g/dL (normal: 12-15.5 g/dL), platelets of 37 × 10^3^/µL (normal: 150-450 × 10^3^/µL), white blood cell count of 19.4 × 10^3^/µL (normal: 4.4-11 × 10^3^/µL) with differentials showing monocytes 11.5 x 10^3^/µL (normal: 0.3 - 0.9 × 10^3^/µL), lymphocytes 3.8 x 10^3^/µL (normal:0.9-2.9 x 10^3^/µL), neutrophils 3.9 × 10^3^/µL (normal: 1.7-7 × 10^3^/µL), and eosinophils 0.1 × 10^3^/µL (normal: 0-0.5 × 10^3^/µL). A comparison of the complete blood count pre-COVID-19 vaccine and post-COVID-19 vaccine is included in Table 1.

**Table 1:** Comparison of pre- and post-COVID-19 vaccine complete blood count

	Before COVID before vaccine (10/2020)	Four days after COVID-19 vaccine - J&J (5/12/21)	One month after COVID vaccine (6/18/21)	Reference values
Hemoglobin	11.8	9.9	6.8	12–15.5 g/dL
Hematocrit	35.8	29.5	20	34.9–44.5%
Red blood cells	3.77	3.01	1.99	3.9–5.03 × 10^6^/µL
White blood cells	6.3	19.4	34.5	4.4–11 × 10^3^/µL
Absolute neutrophils	3.7	3.9	9.6	1.7–7 × 10^3^/µL
Absolute monocytes	0.6	11.5	19.1	0.3–0.9 × 10^3^/µL
Absolute eosinophils	0.2	0.1	0.0	0–0.5 × 10^3^/µL
Absolute lymphocytes	1.8	3.8	5.5	0.9–2.9 × 10^3^/µL
Platelets	199	37	10	150–450 × 10^3^/µL
Reticulocyte count	NA	NA	1.9	0.5–1.5%

The hepatitis panel and human immunodeficiency virus were negative. The thrombocytopenia was concerning for vaccine-related immune thrombocytopenic purpura (ITP). The patient received a tapering dose of steroids and two doses of intravenous immunoglobulin (1 g/kg) as a treatment for ITP, with only a transient rise in platelets. Eventually, the patient developed transfusion-dependent thrombocytopenia. She also started complaining of dry mouth, difficulty swallowing, and new-onset episodes of whitish discoloration of the fingers in cold temperatures. Rheumatologic workup was positive for anti-centromere antibodies and Sojgren’s anti-SSA. C-ANCA, P-ANCA, RF, anti-SSB, and anti-scleroderma 70 were negative. Cryoglobulin, cold agglutinin, and direct coombs were also negative.

On follow-up, it was observed that the patient was progressing to severe anemia and leukocytosis with persistently high and up-trending monocytes. Flow cytometry on peripheral blood and bone marrow biopsies was done to rule out leukemia. Bone marrow biopsy results were significant for chronic myelomonocytic leukemia stage 0 (CMML-0) in a hypercellular marrow. The blasts and promonocytes were not increased; in the setting of severe anemia and thrombocytopenia with monocytosis (AMC 12.6 K/µL - 53.8% of total leucocytes) was consistent with CMML 0. Next-generation sequencing detected KRAS, NPM1, and TET2 gene variations, and karyotyping showed 46, XX female karyotypes. Flow cytometry showed monocytosis (60%) and dysgranulopoiesis with no increased blasts or lymphoproliferative disorder. There is no increase in CD34-positive myeloblasts, and they comprise 0.4% of the total cells. The monocytes (60%) are increased with decreased CD13 and CD14 and increased HLA-DR, suggesting left-shifted maturation. The granulocytes (19%) show decreased side scatter, suggesting hypogranularity with left-shifted CD13/CD16 maturation pattern and aberrant CD56 coexpression, suggesting dysgranulopoiesis. The B-cells (1.7%) are polytypic and the T-cells (8.2%) show a normal CD4:CD8 ratio with no pan T-cell antigen deletion. Viability is 91.13%. The AML panel in the cytogenetics FISH study was negative for RUNX1T1/RUNX1 (ETO/AML1), KMT2A (MLL), PML/RARA, CBFB rearrangement, negative for monosomy 5, and deletion of CSF1R/RPS14 on the long arm of chromosome 5 at q33, negative for monosomy 7, and deletion of MDFIC on the long arm of chromosome 7 at q31. The MPN/CML panel was also negative for BCR/ABL1 rearrangement, trisomy 8, 9, and deletion of DLEU1, DLEU2 on the long arm of chromosome 13 at q14, and deletion of PTPRT on the long arm of chromosome 20 at q12. Risk stratification based on the Mayo Molecular Model classified her as high risk with a score of 3 points and intermediate-risk with the GFM model. Based on the functional status and physical fitness, the patient was deemed to be an intermediate risk but infusion dependent as she has received multiple platelets and pRBC transfusions. As a result, the patient was started on Azacitidine therapy, which significantly improved her cell count after two cycles. However, on further follow-up, it was found that she had progressed to AML and died due to acute respiratory failure secondary to COVID-19 pneumonia.

## Discussion

CMML is a rare and aggressive clonal hematopoietic stem cell neoplasm characterized by monocytosis (≥10% of the white blood cell (WBC) count) [1]. The overlapping clinical, laboratory, and morphologic features of myelodysplasia and myeloproliferation made CMML a subgroup of MDS/MPN in the World Health Organization (WHO) classification of tumors of hematopoietic and lymphoid tissues [1]. Based on the Surveillance, Epidemiology, and End Results (SEER) cancer statistics review 2014-2018, the five-year age-adjusted incidence rate of CMML in both sexes is 0.5/100,000, with a greater incidence in males (0.7/100,000) compared to females (0.3/100,000). [2]. The diagnostic criteria of CMML according to WHO guidelines include persistent peripheral blood monocytosis (≥1 × 10 9/L) with monocytes accounting for ≥10% of the white blood cell (WBC) count for at least three months with the exclusion of all other causes of monocytosis and the presence of <20% blasts in the blood and bone marrow. The disorders that should be excluded include BCR-ABL1+ leukemia, classical MPN, acute myeloid leukemia, and other hematologic malignancies [3]. The presence of mutations associated with CMML like TET2, SRSF2, SETBP1, ASXL1 supports the diagnosis of CMML. Furthermore, CMML is sub-classified into CMML-0, CMML-1, and CMML-2 based on blast cells by WHO and a myelodysplastic variant and a myeloproliferative variant based on white cell count by the French-American-British (FAB) Cooperative Leukemia Group [3,4]. Our patient with 3% blast cells in bone marrow falls under the CMML-0 subgroup. The only disease-modifying treatment option is an allogeneic stem cell transplant. But, most patients remain ineligible given the age of onset and comorbidities. Current therapeutic choice depends on cytopenia-induced or proliferation-associated symptoms, which include erythropoiesis-stimulating agents, cytoreductive agents, and hypomethylating agents [9,10]. There are several ongoing clinical trials on new therapeutic options for CMML. Hypomethylating agents such as azacitidine, decitabine, and oral decitabine/cedazuridine have been approved by the US FDA for the management of CMML [9,10].

The higher prevalence of SIADs has been recognized in CMML patients when compared to the general population. It is estimated that 20% of cases of CMML have an associated SIAD [5]. In a study conducted by Peker et al. on 123 patients with CMML, 24 patients were found to have autoimmune and inflammatory diseases (AID), with ITP being the most common, followed by gout, psoriasis, multiple sclerosis, Sjogren’s syndrome, IBD, autoimmune anemia, and polymyalgia rheumatica, whereas four patients [6] had more than one AID. In another study conducted by Zahid et al. [7] on 377 CMML patients, 77 patients (20%) had associated AID/SIS. This study revealed the heterogeneity of AIDs/SIS associated with CMML, with undefined inflammatory syndromes being the most common, followed by rheumatoid arthritis, psoriasis, immune-mediated thrombocytopenia, polymyalgia rheumatica, and systemic vasculitis. Our patient has scleroderma with elevated centromere 2b antibodies and the symptoms of difficulty swallowing and Raynaud's phenomenon. She also has Sjogren's syndrome, with elevated anti-SSA and dryness of the mouth. We emphasize the fact that, based on the previous studies reported, the association of scleroderma with CMML is very rare. Our patient had concomitant CMML and scleroderma, which were unmasked after the patient received the COVID-19 vaccine.

However, the exact pathogenesis behind this association is yet to be determined. In a systemic review conducted by Ambinder et al. [5], multiple compelling biological theories on the pathogenesis of AIDs in CMML were discussed, which included: (1) chronic inflammation in autoimmune diseases as a trigger for CMML; (2) cytotoxic/immunosuppressive agents used for AIDs as predisposing factors of CMML; (3) AIDs as the consequence of the immune system's response to CMML 4. Clonal myeloid elements trigger AIDs. Based on the aggregate data available, Ambinder et al. support the hypothesis that "SIAIDs are the consequence of CMML.'' Several convincing mechanisms for the above hypothesis include abnormal monocyte behavior and signaling evident in clonal hematopoiesis causing overexpression of CD40 L leading to systemic lupus erythematosis (SLE), overexpression of ICAM-1 and sialic acid-binding Ig-like lectin 1 (Siglec-1), which facilitate the migration of monocytes to peripheral tissue, eventually leading to the increased expression of MHC-II and other costimulatory molecules. Reduced phagocytic activity of monocytes with reduced clearance of apoptotic bodies increases the body's exposure to autoantigens. Genetic aberrations in the clonal myeloid cells can drive SIAIDs, and this can explain the symptoms of SIADs prior to the clinical presentation of CMML [5]. It was found by Zahid et al. that there was no significant difference in the overall survival between patients with AIDs and those without AIDs [7]. On the other hand, Montoro et al. reported poor overall survival among patients with concomitant MDS/CMML and SIAIDs [8]. 

Immunization has always been a life-saving intervention in history. It has saved humanity from several deadly epidemics and pandemics before. As the world is foreseeing the importance of the COVID-19 vaccine, it is essential to report all the possible adverse events for safety monitoring. Our patient had a normal complete blood count at baseline, evident from blood work obtained six months before she got the COVID-19 vaccine. She presented with anemia, thrombocytopenia, and leukocytosis four days after the COVID-19 vaccine. On further follow-up, platelets continued to trend down, even refractory to steroids. Eventually, the patient progressed to transfusion-dependent thrombocytopenia. Persistently elevated monocytes raised the possibility of CMML, which was confirmed after the exclusion of all other possible causes of monocytosis. We suspect that the COVID-19 vaccine has triggered and unmasked both the CMML and the associated scleroderma in our patient, considering the acute onset and absence of other known triggering factors.

## Conclusions

The importance of the COVID-19 vaccine is undeniable during this time. However, reporting possible adverse events helps us to understand the risks that come with it. Our case suggests the possibility of developing CMML associated with limited scleroderma after receiving the J&J COVID vaccine. However, further research has to be done to confirm the hypothesis and to know the pathogenesis behind the association. Physicians should be aware of this unusual complication of the vaccine, and more cases are needed to confirm the association between them.
